# Tumor-targeted delivery of siRNA using fatty acyl-CGKRK peptide conjugates

**DOI:** 10.1038/s41598-017-06381-y

**Published:** 2017-07-21

**Authors:** Meenakshi Sharma, Naglaa Salem El-Sayed, Hung Do, Keykavous Parang, Rakesh Kumar Tiwari, Hamidreza Montazeri Aliabadi

**Affiliations:** 1 Center for Targeted Drug Delivery, Department of Biomedical and Pharmaceutical Sciences, Chapman University School of Pharmacy, Harry and Diane Rinker Health Science Campus, Irvine, California 92618 United States; 2 Cellulose and Paper Department, National Research Center, Dokki, 12622 Cairo Egypt

## Abstract

Tumor-targeted carriers provide efficient delivery of chemotherapeutic agents to tumor tissue. CGKRK is one of the well-known tumor targeting peptides with significant specificity for angiogenic blood vessels and tumor cells. Here, we designed fatty acyl conjugated CGKRK peptides, based on the hypothesis that hydrophobically-modified CGKRK peptide could enhance cellular permeation and delivery of siRNA targeted to tumor cells for effective silencing of selected proteins. We synthesized six fatty acyl-peptide conjugates, using a diverse chain of saturated and unsaturated fatty acids to study the efficiency of this approach. At peptide:siRNA weight/weight ratio of 10:1 (N/P ≈ 13.6), almost all the peptides showed complete binding with siRNA, and at a w/w ratio of 20:1 (N/P ≈ 27.3), complete protection of siRNA from early enzymatic degradation was observed. Conjugated peptides and peptide/siRNA complexes did not show significant cytotoxicity in selected cell lines. The oleic acid-conjugated peptide showed the highest efficiency in siRNA uptake and silencing of kinesin spindle protein at peptide:siRNA w/w ratio of 80:1 (N/P ≈ 109). The siRNA internalization into non-tumorigenic kidney cells was negligible with all fatty acyl-peptide conjugates. These results indicate that conjugation of fatty acids to CGKRK could create an efficient delivery system for siRNA silencing specifically in tumor cells.

## Introduction

Despite significant advances in cancer research and introduction of new generations of molecularly-targeted drugs, cancer is still among leading causes of morbidity worldwide. In fact, more than 14 million of new cases of cancer were diagnosed in 2012, and more than 8 million cancer-related deaths were reported in the same year^1^. American Cancer Society’s estimated number of new cases of breast cancer alone in 2016 is approximately 250,000, and the number of deaths caused by this type of cancer is estimated to exceed 40,000 this year in the US alone^2^. Since the first reports on the possibility of interfering with post-transcription process of protein synthesis using double stranded RNA^3^, RNA interference (RNAi) has been considered a promising alternative approach in cancer therapy. Different approaches to RNAi have been studied, including small interfering RNA (siRNA). Double stranded siRNA is approximately 20–23 nucleotides long, and the antisense strand directs the RNA-induced silencing complex (RISC) to the targeted mRNA. Argonaute 2, a component of RISC, degrades the target mRNA by its ribonuclease activity, and degradation of mRNA suppresses the expression of the target protein^4–7^. siRNA silencing is highly specific for the selected target^8^. Despite advantages of siRNA silencing, there are still several impediments that restrict the use of siRNA in cancer therapy. The main obstacles are the short stability of siRNA under physiological conditions, as siRNA gets readily degraded by the nucleases in the serum^9^, and the negligible cellular internalization due to anionic and hydrophilic nature of the double stranded RNA.

Considering all these barriers, designing a safe and efficient siRNA delivery system is required. Different strategies have been explored for siRNA delivery, which includes specifically designed polymers^10–12^, antibodies^13^, aptamers^14^, lipids^15–17^, and peptides^18, 19^; however, despite occasional promising efficacy *in vitro*, they have mostly proven less than optimal for efficient *in vivo* delivery. Additionally, a promising approach to improve the nucleic acid delivery into the target cells is to incorporate a hydrophobic moiety onto poly cationic polymers^20^. It is reported that, the coupling of different fatty acids with polycationic polymers could increase the ζ-potential towards positive values^12^, and enhance the transfection efficiency of the polymer by improving the ability of the polymer to penetrate the cells, and increase the stability of the nucleotide^21–23^. Since the hydrophobic moiety is believed to increase the vector interaction with the lipophilic cell membrane, it is speculated that increasing the hydrophobicity of the carrier could potentially facilitate cellular uptake. For this study, our goal was to combine the tumor specificity of CGKRK peptide, with the affinity of fatty acids to interact with the cell membrane, and create an effective siRNA carrier.

CGKRK is a tumor homing peptide, discovered by phage display technique. It mediates the cellular internalization in energy and heparin sulfate receptor-dependent manner^24^. Then it localizes to mitochondria in cells. CGKRK peptide exhibited selective high binding affinity to the neovascular endothelial cells and tumor tissues, but not to the non-tumorigenic cells^25, 26^. The unmodified CGKRK peptide has not been studied as the sole carrier for siRNA delivery. Additionally, a promising approach to improve the nucleic acid delivery into cells is to incorporate a hydrophobic moiety to increase permeation to the cellular membrane^7^. Therefore, we hypothesized that the coupling of an optimized fatty acyl chain with CGKRK peptide can improve the ability of the peptide to permeate and deliver siRNA inside the cancer cell to enhance gene silencing efficiency.

**Figure 1:**
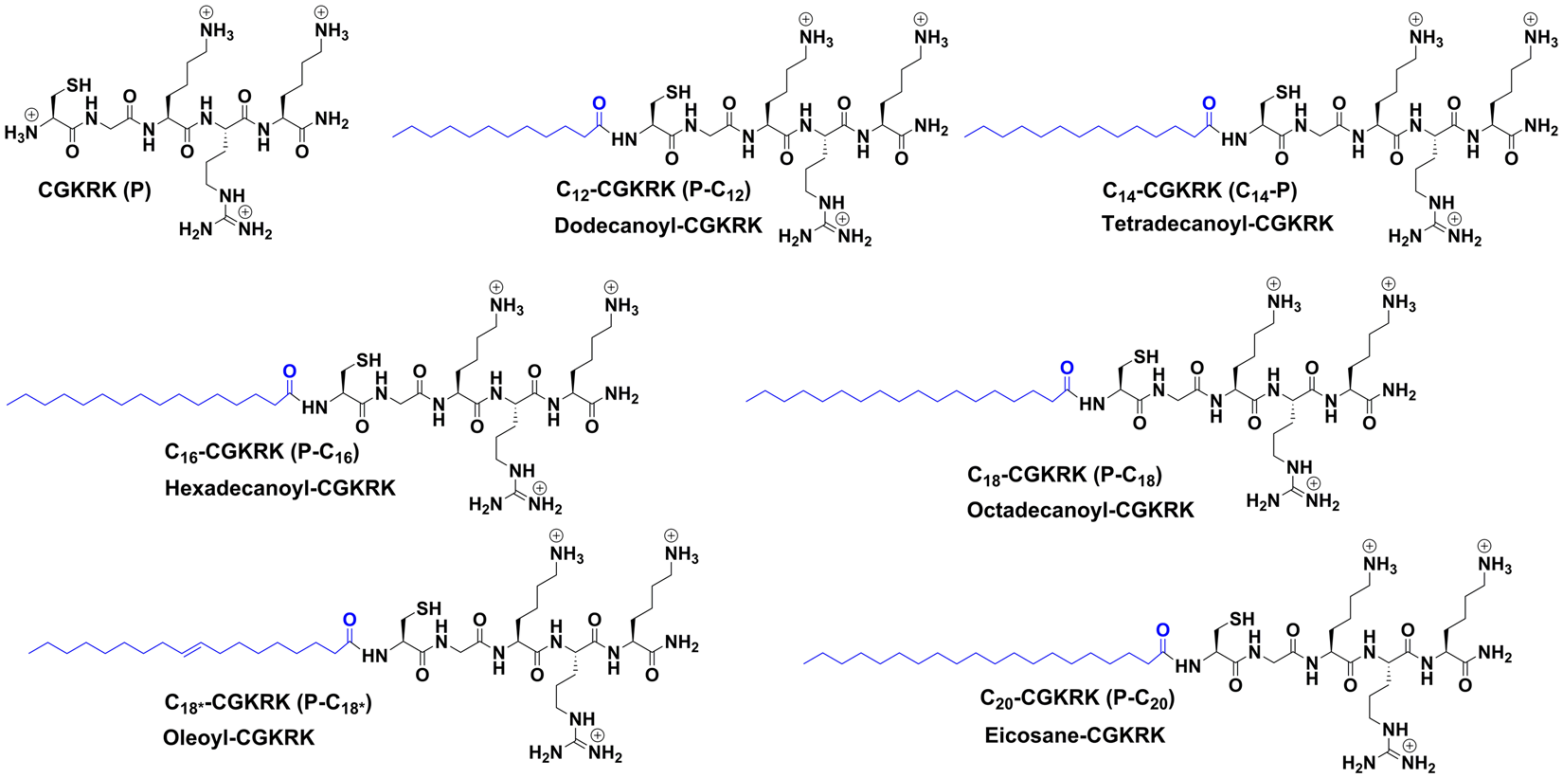
Chemical structures of synthesized fatty acyl-peptide conjugates used in this study.

In this study, we conjugated six different fatty acids with increasing number of methylene (CH_2_) in the chain, namely lauric acid, myristic acid, palmitic acid, oleic acid, stearic acid, and arachidic acid to CGKRK peptide (Fig. 1) and evaluated the impact of the conjugation on the efficiency of the peptide in siRNA delivery to the tumor cells. Fatty acyl-peptide conjugates were purified and characterized using reverse-phase high-pressure liquid chromatography (RP-HPLC) and high-resolution matrix-assisted laser desorption/ionization time-of-flight (MALDI-TOF/TOF) mass spectrometer, respectively. We further characterized and evaluated conjugated peptides by selected parameters i.e. size and zeta potential, siRNA binding, serum stability, specificity for the cancer cells, cellular uptake, and most significantly, the mRNA interference efficacy. To test the conjugated peptides, we used 3 different cancer cells lines (AU-565, a human breast cancer cell line; MDA-MB-435, a human cancer cell line considered a breast cancer cell line for many years, and recently recognized as a melanoma cell line; and MDA-MB-231, a triple negative human breast cancer cell line) and one non-tumorigenic human cell line (human embryonic kidney cells; HEK-293) to observe the specificity of the peptide for the cancer cells.

## Materials and Methods

### Materials

All required organic solvents and fatty acids were purchased from Wilkem Scientific (Pawtucket, RI, USA). Coupling reagents, Rink amide MBHA resin, and Fmoc-amino acid building blocks were purchased from Chem-Impex International Inc. (Wood Dale, IL, USA). 3-(4,5-Dimethylthiazol-2-yl)-2,5-diphenyltetrazolium bromide (catalog # M2128) was from Sigma (St. Louis, MO). Hanks balanced salt solution (HBSS), Dulbecco’s modified Eagle medium (DMEM; low glucose with L-glutamine), RPMI -1640 medium with L-glutamine, fetal bovine serum (FBS), SYBR Green II solution, penicillin (10000 U/mL), Lipofectamine® 2000, and streptomycin (10 mg/mL) were provided by Life Technologies (Grand Island, NY). Scrambled negative control siRNA (catalog # AM4635), 5′-carboxyfluorescein (FAM)-labeled negative control siRNA (catalog # AM4620), and the siRNA targeting kinesin spindle protein (KSP; catalog # AM16704) were obtained from Ambion (Austin, TX). All the primers were purchased from IDT. Heparin sodium salt from porcine intestinal mucosa was purchased from Alfa Aesar (Ward Hill, MA). PCR master mixes iScript^TM^ reverse transcription supermix and iTaq universal SYBR^®^ Green supermix were purchased from Bio-Rad (Hercules, CA). Ethylenediaminetetraacetic acid (EDTA) and all other requirements were provided by Fisher Scientific (Carlsbad, CA).

### Cell lines

The human cancer cell lines, MDA-MB-231, MDA-MB-435, and AU565 were purchased from American Type Culture Collection (ATCC; Manassas, VA). The human embryonic kidney 293 (HEK293) cell line was a gift from Dr. Keykavous Parang’s laboratory. MDA-MB-231 was cultured in DMEM medium, and MDA-MB-435 and AU-565 were grown in RPMI-1640 medium. Both media were supplemented with 10% (v/v) fetal bovine serum, 100 U/mL penicillin and 100 µg/ml streptomycin. Cells were maintained at normal condition of 37 °C and 5% CO_2_ under humidified atmosphere.

### Synthesis and characterization of fatty acyl-peptide conjugates

In general, the peptide was synthesized by the solid-phase synthesis strategy employing *N*-(9-fluorenyl)methoxycarbonyl (Fmoc)-based chemistry and Fmoc-L-amino acid building blocks, under nitrogen at room temperature as described by Oh *et al*.^27^. All the synthesized fatty acyl peptides and the unmodified peptide were purified by reversed-phase Hitachi HPLC (L-2455) equipped with a Waters XBridgeTM BEH130 prep C18 column with OBDTM 10 μm (19 mm × 250 mm). The purification was carried out at 10.0 mL/min flow rate using a gradient of 0−100%, acetonitrile (0.1% TFA) and water (0.1% TFA) over 60 min. High-resolution MALDI-TOF/TOF mass spectrometers (ABX SCIEX TOF/TOF and Bruker autoflex speed MALDI-TOF/TOF) were used to confirm the structure of the peptide conjugate in the purified fraction. A solution of α-cyano-4-hydroxycinnamic acid (5 mg/ml) in acetonitrile (0.1% TFA) and water (0.1% TFA) (1:1, v/v) was freshly prepared as the matrix. An aliquot of the sample was mixed with the matrix at the ratio of 1:3 and spotted onto the MALDI plate, and was air dried in the dark for few min before the analysis. The purified peptide fractions were pooled, evaporated, and kept in −80 °C refrigerator overnight and then lyophilized for 48 h to get powdered peptide for further assays. The lyophilized peptides were stored in −20 °C refrigerator until their use in the assay. The mass of the compounds was confirmed by high-resolution MALDI- TOF/TOF mass spectrometers.

**Figure 2:**
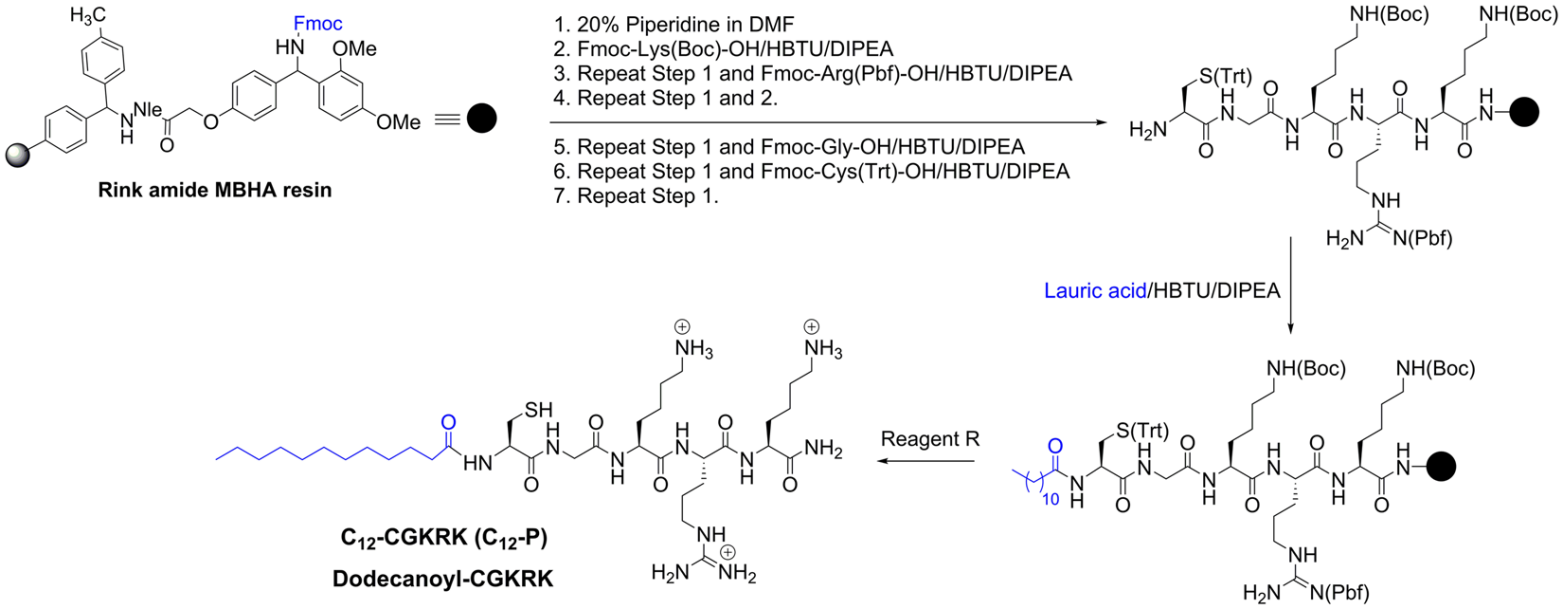
Synthesis of Dodecanoyl-CGKRK as a Representative Example.

As a representative example, the synthesis of dodecanoyl-CGKRK is described here (Fig. 2). The linear peptide sequence CGKRK was synthesized using Tribute automated peptide synthesizer (Protein Technology, Inc., Arizona), in 0.40 mmol scale. The Rink amide MBHA resin (loading 0.52 mmol/g, 770 mg) was swelled for approximately 30 min under anhydrous DMF with the help of dry nitrogen in glass vessel fitted with frit (manual peptide synthesis vessel). All coupling and deprotection steps were carried out at room temprature. The solvent was filtered off using a vacuum, and the swelling and filtration steps were repeated two more times before resin was transferred to the tribute peptide synthesizer vessel to start automated peptide synthesis. To assemble linear peptide sequence, the synthesis was started using building block amino acids containing side chain and Fmoc protection, such as Fmoc-Lys(Boc)-OH (468 mg, 1 mmol), Fmoc-Arg(Pbf)-OH (648.77 mg, 1 mmol), Fmoc-Lys(Boc)-OH (468 mg, 1 mmol), Fmoc-Gly-OH (297.31 mg, 1 mmol), and Fmoc-Cys(Trt)-OH (585.71 mg, 1 mmol), using DMF as solvent and 2-(1*H*-benzotriazole-1-yl)-1,1,3,3-tetramethyluranium hexafluorophosphate (HBTU, 1 mmol), and 0.4 M *N*-methyl morpholine in DMF as coupling, and activating reagents, respectively. The Fmoc deprotection at each step was carried out using piperidine in DMF (20% v/v). After the linear peptide was assembled, the peptidyl-resin was transferred to a manual peptide synthesis vessel. A solution of lauric acid (200 mg, 1 mmol, dissolved in DCM/DMF mixture) was added to the mixture, and then activated using HBTU (379 mg, 1 mmol) and DIPEA (4 molar equivalent, 697 µL) with vortexing for 10 min. The mixture was agitated using nitrogen for 24 h at room temperature. Afterward, the resin was washed several times with DMF (3 times, 10 min each), DCM (3 times, 10 min each), and acetone (3 times, 10 min each) and dried. The peptide was cleaved from the resin, and the side chains protection were removed by agitating in freshly prepared cleavage cocktail using “Reagent R” (20 mL, TFA:thioanisole:1,2-ethanedithiol:anisole, 92:5:3:2, v/v/v/v) at room temperature for 6 h. The crude dodecanoyl-CGKRK was precipitated by adding cold diethyl ether (Et_2_O, 50 mL × 2 times) and centrifuged for 10 min. The precipitate was collected, dissolved in acetonitrile (0.1% TFA) with the help of few drops of water (0.1% TFA), and purified by reversed-phase Hitachi HPLC (L-2455) equipped with a Waters XBridgeTM BEH130 prep C18 column with OBDTM 10 μm (19 mm × 250 mm). The purification was carried out at 10.0 mL/min flow rate using a gradient of 0−100%, acetonitrile (0.1% TFA) and water (0.1% TFA) over 60 min. High-resolution MALDI-TOF/TOF mass spectrometers (ABX SCIEX TOF/TOF and Bruker autoflex speed MALDI-TOF/TOF) were used to confirm the structure of the peptide conjugate in the purified fraction. An aliquot of the sample was mixed with the matrix at the ratio of 1:3 and spotted onto the MALDI plate, and was air dried in the dark for few min before the analysis. The purified peptide fractions were pooled, evaporated, and keept in −80 °C refrigerator over night and then lyophilized for 48 h to get powdered peptide for further assays.

**CGKRK (P):** MALDI-TOF (m/z): C_23_H_47_N_11_O_5_S calcd. 589.3482; found 590.6109 [M + H]^+^; **Dodecanoyl-CGKRK (P-C12):** MALDI-TOF (m/z): C_35_H_69_N_11_O_6_S calcd. 771.5153; found 772.2769 [M + H]^+^; **Tetradecanoyl-CGKRK (P-C14):** MALDI-TOF (m/z): C_37_H_73_N_11_O_6_S calcd. 799.5466; found 800.2928 [M + H]^+^; **Hexadecanoyl-CGKRK (P-C16):** MALDI-TOF (m/z): C_39_H_77_N_11_O_6_S calcd. 827.5779; found 828.2538 [M + H]^+^; **Octadecanoyl-CGKRK (P-C18):** MALDI-TOF (m/z): C_41_H_81_N_11_O_6_S calcd. 855.6092; found 857.7393 [M + 2 H]^+^; Oleoyl-CGKRK (P-C18*): MALDI-TOF (m/z): C_41_H_79_N_11_O_6_S calcd. 853.5936; found 854.3768 [M + H]^+^; **Eicosane-CGKRK (P-C20)**: MALDI-TOF (m/z): C_43_H_85_N_11_O_6_S calcd. 883.6405; found 884.3861 [M + H]^+^.

### Peptide/siRNA complex formation

Peptide/siRNA complexes were formed by simple mixing of siRNA and peptides in different w/w ratios, as described in our previous reports^11, 12^. First, siRNA was added to 150 mM NaCl solution. The fatty acid-conjugated peptide was dissolved in double-distilled water at a stock concentration of 2 mg/mL. The peptide solution was added to siRNA/saline mixture at different proportions, and mixed. The complexes were incubated at room temperature for 30 min for complete complex formation. The complexes were formed using pre-decided peptide:siRNA ratios, which will be specified during the manuscript as both weight/weight and N/P ratios. Where w/w ratio was simply calculated by dividing the total weight of peptide present in the complex solution added to the cells to the weight of siRNA in the same solution, N/P ratio was calculated by equation 1:

1 $$\frac{N}{P}=\frac{(Number\,of\,moles\,for\,peptide\times Number\,of\,Nitrogens)}{Number\,of\,moles\,for\,siRNA\times 48}$$

where 48 represents the average number of phosphates in each siRNA molecule. The N/P ratios mentioned throughout the manuscript is the average of N/P ratios for each individual modified peptide.

### Size and ζ-potential of nanoparticles

Peptide/siRNA complexes were prepared with peptide:siRNA weight/weight ratio of 1:1 (N/P ≈ 1.4), 5:1 (N/P ≈ 6.8), 10:1 (N/P ≈ 13.6), and 20:1 (N/P ≈ 27.3). Size and zeta (ζ) potential of the complexes were measured at least three times each using a Malvern Nano ZS Zetasizer (Westborough, MA). All measurements were performed at 25 °C. Zeta potential of the complexes was determined in a disposable folded capillary cell at a voltage of 40 V using Smoluchowski approximation.

### Binding affinity for siRNA

The binding affinity of the peptide to siRNA was investigated by SYBR Green II dye exclusion assay, as we have reported previously^12^. Briefly, scrambled siRNA solutions were prepared in RNase-free water (0.5 μg/ml), and peptide/siRNA complexes were prepared with w/w ratios of 0.125:1 (N/P ≈ 0.17), 0.317:1 (N/P ≈ 0.43), 0.625:1 (N/P ≈ 0.85), 1.25:1 (N/P ≈ 1.7), 2.5:1 (N/P ≈ 3.4), 5:1 (N/P ≈ 6.8), and 10:1 (N/P ≈ 13.6) (with free siRNA as control). After 30 min incubation at room temperature, 200 μl of SYBR Green II solution (1:10,000 dilution in water) was added to the complexes. The fluorescence of the samples was quantified in a 96-well black plates (λ excitation: 485 nm, λ emission: 527 nm) to determine the proportion of unbound siRNA. The percentage of siRNA bound to the peptide was plotted against the peptide/siRNA ratio, and the ratio required for 50% binding (BC50) was calculated based on the line equation of the linear portion of the curve under these experimental conditions.

### Serum stability of peptide-bound siRNA

Complexes of peptide/siRNA were prepared using scrambled siRNA and the synthesized peptides with the w/w ratios of 5:1 (N/P ≈ 6.8), 10:1 (N/P ≈ 13.6), 20:1 (N/P ≈ 27.3), and 40:1 (N/P ≈ 54.6), using free siRNA as a positive control. Complexes were incubated in triplicates at 37 °C in a 25%v/v FBS solution in HBSS for 24 h. At the end of incubation period, heparin (2 µl of 5% solution in normal saline) and EDTA (3 µl of 0.5 mM solution) were added to the samples for complete dissociation of the siRNA from the complexes. The samples were analyzed on a 1% agarose gel containing 1 µg/mL ethidium bromide at 70 V for 20 min. The gel was visualized under UV illumination (Gel-Doc system, Bio-Rad; Hercules, CA), and the intensity of the bands corresponding to the remaining intact siRNA was quantified using Image J software.

### Cytotoxicity of peptides and peptide/siRNA complexes

The cytotoxicity of the delivery system was investigated in different human cancer cell lines (AU565, MDA-MB-231, and MDA-MB-435) and a human non-tumorigenic kidney cell line (HEK293 cells) after exposure to peptides alone, or in complex with siRNA. Confluent cell cultures (~600,000–800,000 cells/mL) were trypsinized and seeded in 96 well plates with 100 µl of the medium in each well, and allowed to reach ~50% confluency in 24 h. Peptides were added to the wells at a final concentration of 20 µg/mL, 40 µg/mL, and 80 µg/mL in triplicate. Peptide/siRNA complexes were prepared using scrambled siRNA (final concentration of 36 nM in cell media) at a w/w ratio of 20:1 (N/P ≈ 27.3), 40:1 (N/P ≈ 54.6)) and 80:1 (N/P ≈ 109) (which translate to 10.3, 20.6, and 41.2 µg/mL final concentration, respectively). After 72 h incubation at 37 °C and regular cell culture conditions, 20 µl of 3-(4,5-dimethylthiazol-2-yl)-2,5-diphenyltetrazolium bromide (MTT) solution (5 mg/mL in HBSS) was added to each well. After 45 min of incubation at 37 °C, the medium was removed, and the formazan crystals that formed were dissolved in 100 µl of DMSO per well. The absorbance at 570 nm was measured with Spectra Max M5 plate-reader (Molecular Devices; Sunnyvale, CA) with no dye added to three wells as blank. The relative cell viability was calculated and expressed as a percentage relative to untreated cells.

### Cellular Internalization

Confluent cell cultures were seeded in 24-well plates at ~200,000 cells/mL. Peptide/siRNA complexes were prepared using FAM-labeled scrambled siRNA with peptide:siRNA w/w ratios of 20:1 (N/P ≈ 27.3), 40:1 (N/P ≈ 54.6)) and 80:1 (N/P ≈ 109) (corresponding to 36 nM siRNA and ~10, 20 and 40 µg/mL peptide in culture media, respectively) and were added to the cells after 24 h of seeding. After 24 h of incubation at 37 °C, the cells were washed with HBBS, trypsinized, and fixed with 3.7% formaldehyde solution. The siRNA uptake was quantified by Flow cytometer (BD-FACSVerse, BD Biosciences; San Jose, CA) using FITC channel to detect the cell-associated fluorescence signal. The percentage of cells showing FAM fluorescence and the mean fluorescence in the selected cell population were determined. Calibration was performed by gating with the negative control (i.e., “No Treatment”) group, so that the auto-fluorescent cells represented ~1% of the total cell population.

For fluorescence microscopy, cells were seeded in 24 well plates at ~400,000 cells/mL. After 24 h, similar peptide/siRNA complexes (prepared at a peptide:siRNA w/w ratio of 80:1 (N/P ≈ 106 with P-C18*) were added to the cells. After 24 h of incubation at 37 °C, the cells were washed with HBBS and fixed with 3.7% formaldehyde. Cell membrane and nucleus were stained with Texas Red Phalloidin (Invitrogen) (1:250 in HBSS) and DAPI and incubating the cells for 1 h and 5 min, respectively and consecutively, at room temperature, followed by washing with HBSS. Keyence BZ-X700 all-in-one Fluorescence Microscope (KEYENCE Corp. of America, Itasca, IL) was used to take fluorescence images using the 20x objective and different filters for DAPI, FITC, and Texas Red. Untreated cells and cells exposed to free siRNA were used as negative control.

### Interference with model mRNA and Real-Time PCR

Confluent AU565 cell cultures were seeded in 6-well plates at ~400,000 cells/mL for mRNA analysis by real-time PCR. Peptide:siRNA complexes were prepared using scrambled siRNA (as a negative control) or KSP siRNA, with a w/w ratio of 80:1 (N/P ≈ 109) and final siRNA concentration of 54 nM. As a positive control, a group of cells were exposed to the same concentration of siRNA delivered by Lipofectamine® 2000, using the instructions provided by the manufacturer. Complexes were added to the cells, and cells were incubated for 48 h at 37 °C. Cells were then lysed with TRIzol (1 mL for each 1 × 10^6^ cells) and incubated at room temperature for 5 min. Chloroform (0.2 mL for each mL of TRIzol) was added to the lysates, and the aqueous phase was separated after shaking and 2–3 min incubation at room temperature. Isopropanol was used to precipitate RNA and the pellet was washed with 75% ethanol. Extracted RNA was then dissolved in RNase-free water, and the total RNA was determined by BioSpec-Nano (Shimadzu, Columbia, MD). RNA (0.5 µg) was reverse transcribed to synthesize cDNA using iScriptTM reverse transcription supermix and the C1000 Touch^®^ thermocycler (Bio-Rad, Hercules, CA) according to the manufacturer’s guidelines. A CFX96^TM^ optical module (Bio-Rad, Hercules, CA) was used for RT-PCR analysis using human β-actin as the endogenous gene. Primers were designed using primer Blast software available at The National Center for Biotechnology Information website (http://www.ncbi.nlm.nih.gov/) and were synthesized with the following sequences by the IDT Technologies (Coralville, Iowa): β- Actin (Forward: 5′-CCA CCC CAC TTC TCT CTA AGG A-3′; Reverse: 5′-AAT TTA CAC GAA AGC AAT GCT-3′) and KSP (Forward: 5′-TCA CAA AAG CAA TGT GGA AAC CTA-3′; Reverse: 5′-TCT GTC CAA AGA TTCA TTA ACT TGC A-3′). Primers were tested to assure equal efficiency at different cDNA concentrations (with a slope < 0.1 for the ΔCT vs. cDNA dilution graph) and selectivity for the mRNA of interest. Analysis was performed by calculating ΔCT, ΔΔCT, and relative quantity (RQ) using endogenous gene and “no treatment” group as reference points.

### Statistical methods

The significance of the differences observed in siRNA uptake, mRNA levels (analyzed by RT-PCR), and cell viability were evaluated using Student’s t-test (with p < 0.05 considered significant). Two-way analysis of variance (ANOVA) was used to compare multiple groups followed by Bonferroni post hoc test. Standard deviations were calculated for all results shown and are represented by the error bars in all figures. Pearson’s correlation coefficient was calculated where indicated, and its significance was determined by the t-test, according to the equation 2:

2 $$t=r\sqrt{\frac{n-2}{1-{r}^{2}}}$$

where r and n are the correlation coefficient and the number of samples, respectively. The calculated value of t was compared to p values for each degree of freedom to determine the significance of the correlation.

## Results

### Synthesis and characterization of fatty acyl-CGKRK peptides

The peptides used in the study (Fig. 1) were synthesized using Fmoc/tBu solid phase chemistry. After assembly of CGKRK peptide, fatty acyl chains with various length (C12–C20) were incorporated at *N*-terminal during solid-phase peptide chemistry, following the method described by Oh *et al*.^27^, using commercially available fatty acids namely lauric acid (saturated acid, C-12), myristic acid (saturated acid, C-14), palmitic acid (saturated acid, C-16), stearic acid (saturated acid, C-18), oleic acid (unsaturated fatty acid, C-18), and arachidic acid (saturated acid, C-20) and HBTU/DIPEA as coupling and activating reagents, respectively. Fatty acyl conjugated peptide were cleaved from the solid support using freshly prepared cleavage cocktail reagent R followed by purification using HPLC and characterized by MALDI mass spectrometry (See **Supporting Information**). The purity of peptide was found to be > 95%. As a representative example, the synthesis of dodecanoyl-CGKRK conjugate is shown in Fig. 2.

### Size and surface charge of the Peptide-siRNA complexes

**Figure 3:**
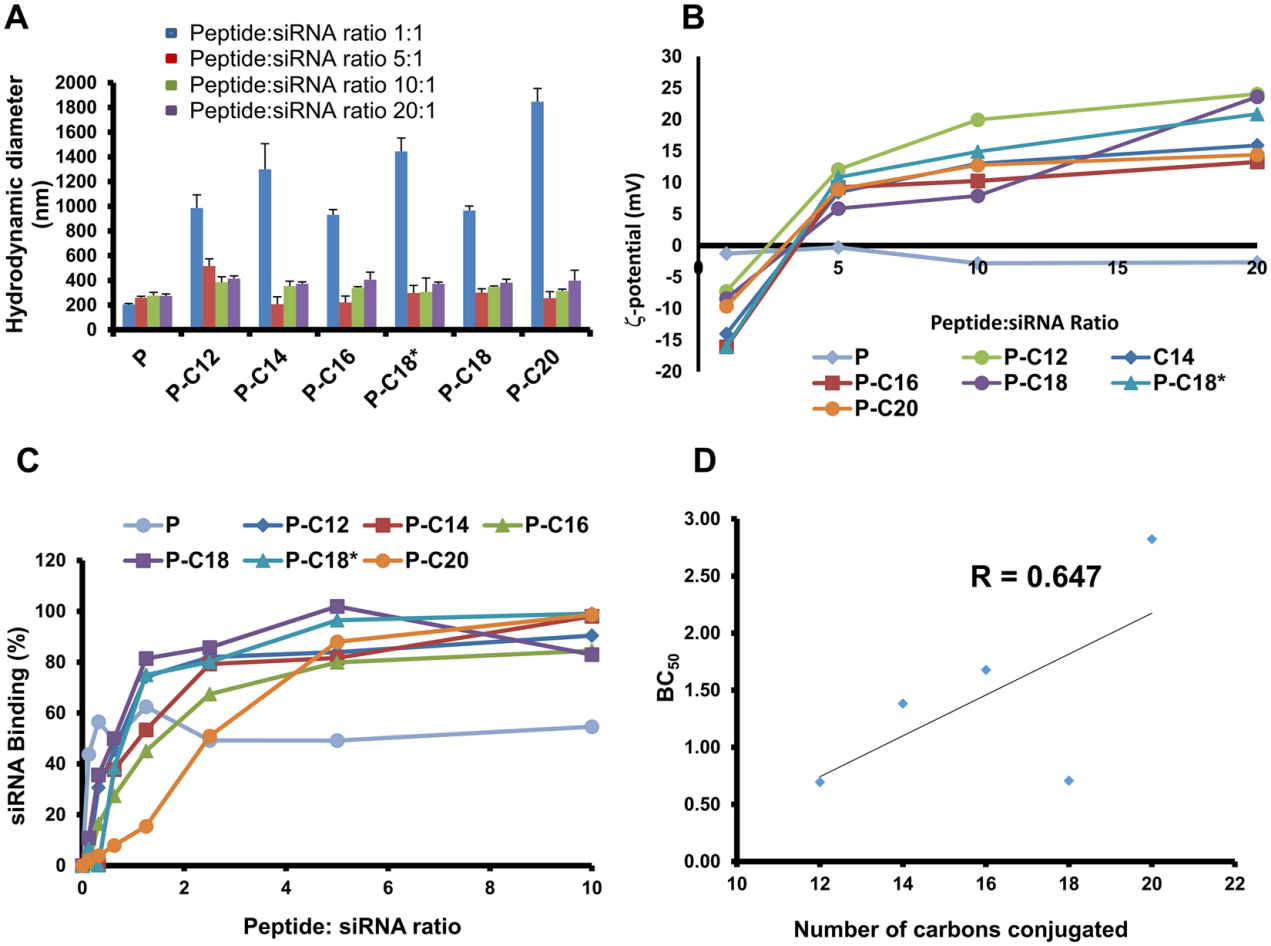
Size, ζ-potential, and binding affinity of designed peptides. (**A**) Hydrodynamic diameter of native peptide (CGKRK) and the fatty acid conjugated peptides, analyzed by light scattering. A significant drop in the size of particles with increasing the peptide:siRNA w/w ratio to 5:1 (N/P ≈ 6.8) indicates the complex formation; (**B**) ζ-potential of the unmodified and the fatty acid-conjugated peptides. A gradual shift to positive surface charge also indicates complex formation with modified peptides. Similar trend was not observed for unmodified CGKRK; (**C**) Representative graph of Binding affinity of the peptide library to scrambled siRNA, indicating the percentage of siRNA bound to the peptide in different peptide:siRNA w/w ratios. Complete binding was observed for all modified peptides at ratio of 5:1 (N/P ≈ 6.8); (**D**) Correlation between the peptide:siRNA complex ratios required for 50% binding (BC_50_) and the size of the fatty acid conjugation. R = 0.647 indicated a moderate correlation; however, the correlation coefficient was not significant in Pearson correlation coefficient analysis.

The effect of peptide structure and peptide:siRNA ratio on the hydrodynamic size and surface charge of the particles were analyzed. The results are summarized in Fig. 3A and B. The size of most of the peptide/siRNA complexes were in the range of 200 to 400 nm, with the exception of the peptide/siRNA ratio of 1:1 (N/P ≈ 1.4) that resulted in larger complexes (>800 nm) for all of the fatty acyl-peptide conjugates. Particles formed with the unmodified peptide were relatively small (180–220 nm). A specific trend in the complex sizes based on the chain length of the fatty acid was not observed (Fig. 3A). The siRNA complexes formed with the unmodified peptide showed negative ζ-potential, even at the highest studied peptide/siRNA ratio (20:1; N/P ≈ 27.3). The fatty acyl-substituted peptides, however, showed a constant increase in the ζ-potential values, and all had a positive surface charge at peptide:siRNA ratios of 5:1 (N/P ≈ 6.8) and higher (Fig. 3B). A significant difference was not detected between the surface charges of particles formed with different lipid-substituted peptides, however, the smallest substituted fatty acid (lauric acid, C12) seemed to show higher positive values in ratios of 5:1 (N/P ≈ 6.8) and higher.

### Binding Affinities

The affinity of each peptide to bind to siRNA and form nanoscale complexes was determined by quantifying SYBR green fluorescence (which is magnified as it binds to free siRNA) in triplicate, and the results are summarized in Fig. 3C. Among the studied peptides, the unmodified peptide did not achieve complete binding (even at a peptide:siRNA ratio of 10:1; N/P ≈ 13.6), and the siRNA binding percentage plateaued at ~50%. The fatty acyl-conjugated peptides, on the other hand, showed almost complete binding at the peptide:siRNA ratio of 5:1 (N/P ≈ 6.8). After calculating BC50 for the studied peptides, we analyzed the correlation between the number of methylenes (CH_2_) in the acylated peptide (representing the hydrophobicity of the conjugates) and the BC50, which is illustrated in Fig. 3D. While the BC50 for the arachidic acid-conjugated peptide (C20; the largest fatty acid used) was significantly higher than the other peptides (indicating a lower affinity), and that seemed to be the overall trend, the correlation was not significant (P < 0.1). There was no significant difference in the BC50 calculated for C18 saturated and unsaturated conjugations (stearic and oleic acids, respectively).

### Stability against Enzymatic Degradation

**Figure 4:**
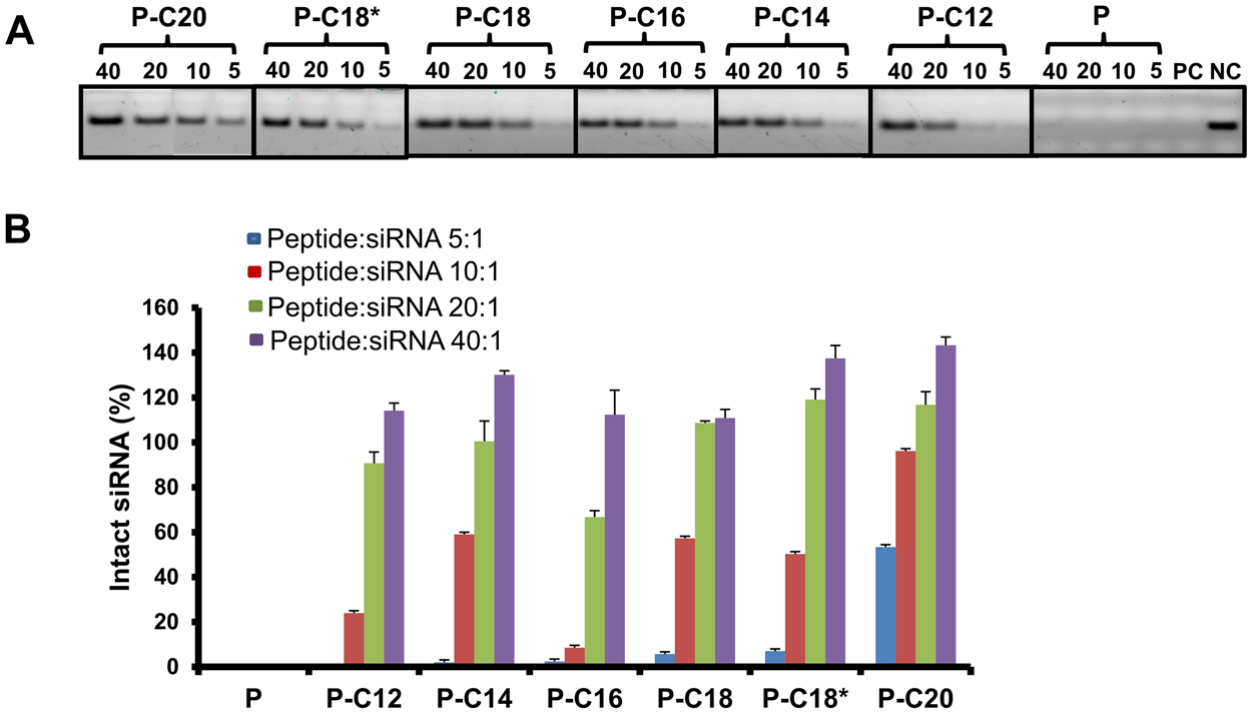
Serum stability of peptide:siRNA complexes. (**A**) Representative gel electrophoresis image of intact siRNA remaining after 24 h incubation with 25% FBS at 37 °C. PC = Positive Control representing free siRNA incubated with serum; NC = Negative Control (Quantified as 100%) representing free siRNA incubated with saline; (**B**) Quantification of intact siRNA remaining after 24 h incubation. Complete protection was observed for all modified peptides at w/w ratio of 20:1 (N/P ≈ 27.3), except for P-C16, which achieved that at ratio of 40:1 (N/P ≈ 54.6).

Due to the extreme sensitivity of siRNA to enzymatic degradation (mostly via RNase), we analyzed the stability of siRNA after 24 h exposure to FBS, to determine the protection afforded by the peptides. Free siRNA exposed to saline and FBS were used as negative and positive controls, respectively. Heparin was added to the complex after the incubation period, to release siRNA from the complexes, and the percentage of intact siRNA (compared to negative control) was determined after for peptide/siRNA ratios of 5:1, 10:1, 20:1, and 40:1 (N/P ratios of 6.8, 13.6, 27.3, and 54.6, respectively) using gel electrophoresis (Fig. 4). Free siRNA was completely degraded, after the incubation period (Fig. 4A). The unmodified peptide did not show any protection, even at a highest ratio of 40:1 (N/P ≈ 54.6), as there was no intact siRNA detected after 48 h incubation. With peptide/siRNA ratio of 20:1 (N/P ≈ 27.3) all fatty acid-substituted peptide showed almost complete protection against degradation except for palmitic acid-conjugated peptide (P-C16; ~66% siRNA detected after 24 h compared to negative control). At peptide:siRNA ratio of 5:1 (N/P ≈ 6.8), none of the fatty acylated peptides showed more than 10% siRNA remaining, except for arachidic acid-conjugated peptide (P-C20), where we detected ~53% of siRNA remaining. The level of intact siRNA left, constantly increased with the increase in peptide:siRNA ratio for all the studied peptides (Fig. 4B).

### Cytotoxicity of Delivery System

**Figure 5:**
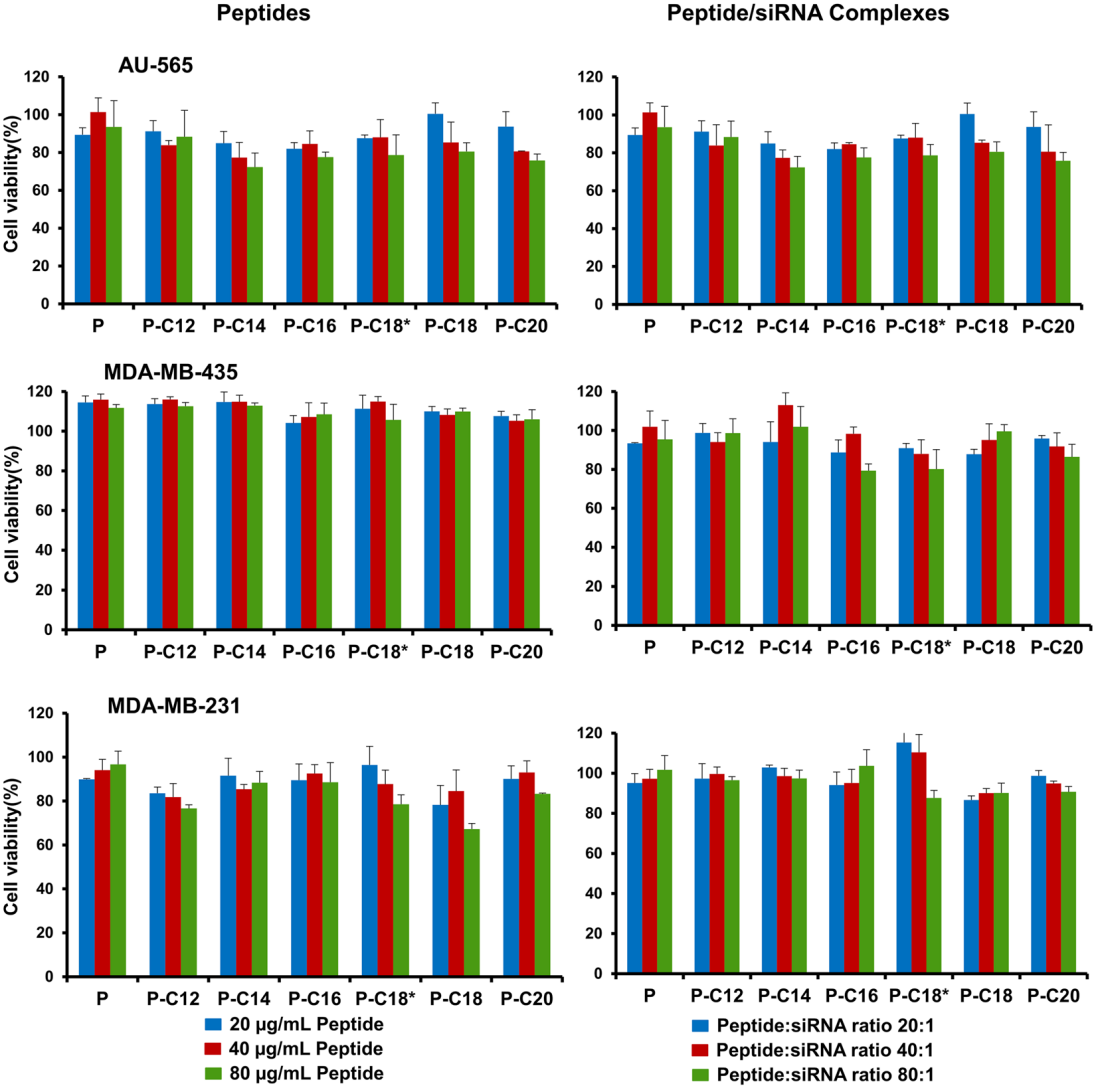
Cellular toxicity of peptides (left panels) and peptide/siRNA complexes (right panels). The viability of AU-565 cells, MDA-MB-435, and MDA-MB-231cells was evaluated using MTT assay after 72 h exposure to different concentration of peptides (left panels), or to complexes formed with scrambled siRNA and peptides at different peptide:siRNA w/w ratios. The percentage of viability is presented as Mean + SD; n = 3). No significant toxicity was observed for any of the studied peptides. For the similar experiment in HEK293 cells, see Supplementary Figure 1.

The toxicity of the peptides was determined by two approaches: exposing MDA-MB-231, MDA-MB-435, AU-565 (human cancer cell lines), and HEK293 cells (human non-tumorigenic kidney cells) to peptides, or peptide:siRNA complexes. The results are summarized in Fig. 5. We investigated the effect of a wide range of peptide concentrations and peptide:siRNA ratios on cell viability after 72 h of exposure. In all studied groups, ~80% or more of the cells were viable, even with highest concentration of peptide alone, or as a complex with siRNA. The only exception was the cells treated with 80 µg/mL of the stearic acid-conjugated peptide in MDA-MB-231 cells, which demonstrated ~67% viability. A similar effect was observed for this peptide in HEK293 cells (Supplementary Figure 1). No particular trend was detected in the toxicity of the peptides with the alteration of the conjugated fatty acid. Also, a significant difference was not detected between the overall toxicity of the unmodified or modified peptides in different cell lines. Moreover, forming complex with siRNA did not have a significant effect on the toxicity of the studied peptides in any of the included cell lines.

### Intracellular Delivery of siRNA

**Figure 6:**
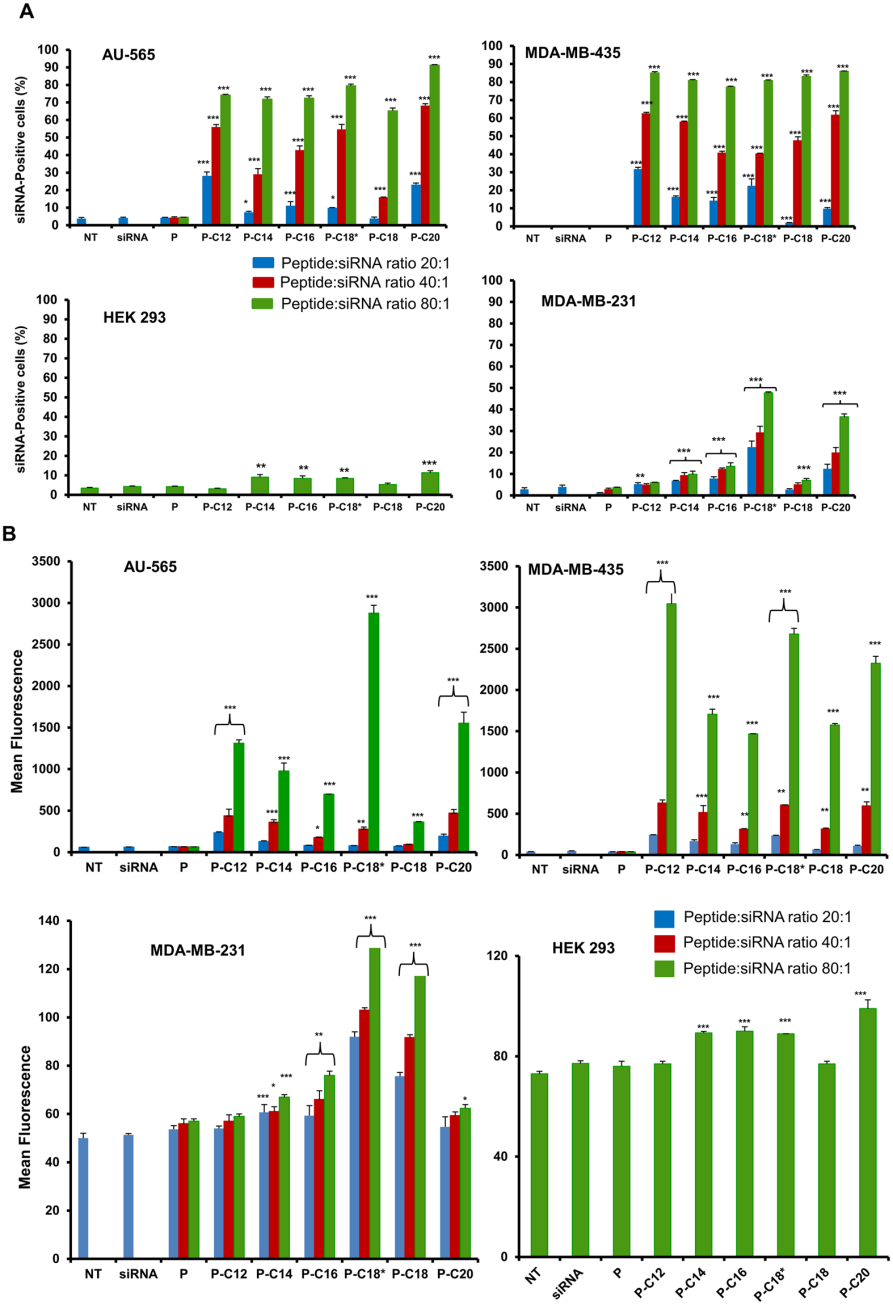
Cellular uptake of FAM-labelled siRNA. (**A)** The percentage of siRNA-positive cells for all the studied peptides at different peptide:siRNA w/w ratios. Modified peptides showed a significantly enhanced uptake compared to unmodified CGKRK, which continuously increased with higher ratios. No significant uptake was observed in normal HEK293 cells; (**B**) The extent of cellular internalization is also presented as the mean fluorescence of the whole cell population, which demonstrates the differences in siRNA uptake more efficiently. Overall, the extent of uptake was lower in the MDA-MB-231 cells compared to the other cancer cells included in this experiment. Uptake in HEK293 cells was negligible. In all graphs, data is shown as Mean + D (n = 3). ***p < 0.001; **p < 0.01; and *p < 0.05; calculated by the 2-way ANOVA with Bonferroni post-test using Graph pad Prism software.

To analyze the impact of fatty acid conjugation to the CGKRK peptide on the siRNA delivery efficiency, different cancer cell lines were used to evaluate fluorescent-labeled siRNA internalization after complex formation with the unmodified and synthesized peptides. The results were evaluated in three cancer cell lines (AU-565, MDA-MB-231, and MDA-MB-435), and a non-tumorigenic human cell line (HEK293) as mean fluorescence and percentage of cells positive for the fluorescence signal, as summarized in Fig. 6. The unmodified peptide did not create a significant difference in siRNA delivery compared to free siRNA, neither of which were statistically different from not treated (NT) cells. In contrast, the fatty acid-conjugated peptides showed efficiency in siRNA cellular internalization that progressively increased with an increase in peptide:siRNA ratio. At the highest ratio studied (80:1; N/P ≈ 109), more than 75% of MDA-MB-435 cells and more than 65% of AU-565 cells were fluorescent-positive for all modified peptides. Arachidic acid-conjugated peptide (P-C20) showed the highest percentage of siRNA-positive cells in AU-565 with more than 90% uptake. The 2-way ANOVA for AU-565 cell line showed the siRNA uptake ranking of: P-C18 < P-C12 = P-C14 = P-C16 < P-C18* < P-C20. In MDA-MB-435 cells, siRNA uptake exceeded 85% with both lauric acid and arachidic acid-conjugated peptides, and the ranking for modified peptides was: P-C16 < P-C18* = P-C14 < P-C18 = P-C12 = P-C20. The overall percentage of fluorescent-positive cells was significantly lower in MDA-MB-231 cells, where only oleic acid and arachidic acid-conjugated peptides (P-C18* and P-C20, respectively) were able to internalize siRNA into ~48% and ~37% of the cells, respectively (Fig. 6A). The siRNA internalization efficiency ranking among all modified peptides in MDA-MB-231 cells was P-C12 = P-C18 < P-C14 < P-C16 < P-C20 < P-C18*. No significant correlation between the number of CH_2_ groups in the conjugated fatty acid and the percentage of fluorescent positive cells was observed (data not shown).

The mean fluorescence of the cell population followed a similar trend, and the highest uptake was achieved with peptide:siRNA ratio of 80:1 (N/P ≈ 109) for each of the modified peptides, with significantly lower values for MDA-MB-231 cells. However, the mean fluorescence better revealed the differences in delivery performance among the modified peptides (Fig. 6B). In AU-565 cells, oleic acid-conjugated peptide (P-C18*) created the highest mean fluorescent (~2900), and the ranking of the modified peptides in this cell line was: P-C18 < P-C16 < P-C14 < P-C12 < P-C20 < P-C18*. The overall pattern in the mean fluorescence in MDA-MB-435 cells was somewhat similar (with P-C12, P-C18*, and P-C20 demonstrating the highest mean fluorescent); however, the highest value was observed for lauric acid-conjugated peptide (~3050), and the overall ranking in this cell line was: P-C16 < P-C18 < P-C14 < P-C20 < P-C18* < P-C12. As mentioned before, the overall mean fluorescence values in MDA-MB-231 cells were significantly lower, and the highest value was observed with P-C18* (~132). In this cell line, the modified peptides ranked as: P-C12 = P-C20 = P-C14 < P-C16 < P-C18 < P-C18*.

Since CGKRK is a tumor homing peptide, we compared the siRNA uptake by cancer cell lines with the non-tumorigenic embryonic kidney cell line (HEK293). Only the highest ratio was studied for this peptide, and even though some of the percentages of fluorescent-positive cells and mean fluorescence values were significantly different from the unmodified peptide, no significant difference was observed between “No Treatment”, free siRNA, and unmodified peptide. Similarly, a significant correlation was not observed between number of carbons in the conjugated fatty acid and the generated mean fluorescent values (data not shown). The highest percentage of fluorescent-positive cells and the highest mean fluorescence value observed in this non-cancerous cell line were ~9% and 99% (were free siRNA created mean fluorescence of 77%), respectively (Fig. 6).

**Figure 7:**
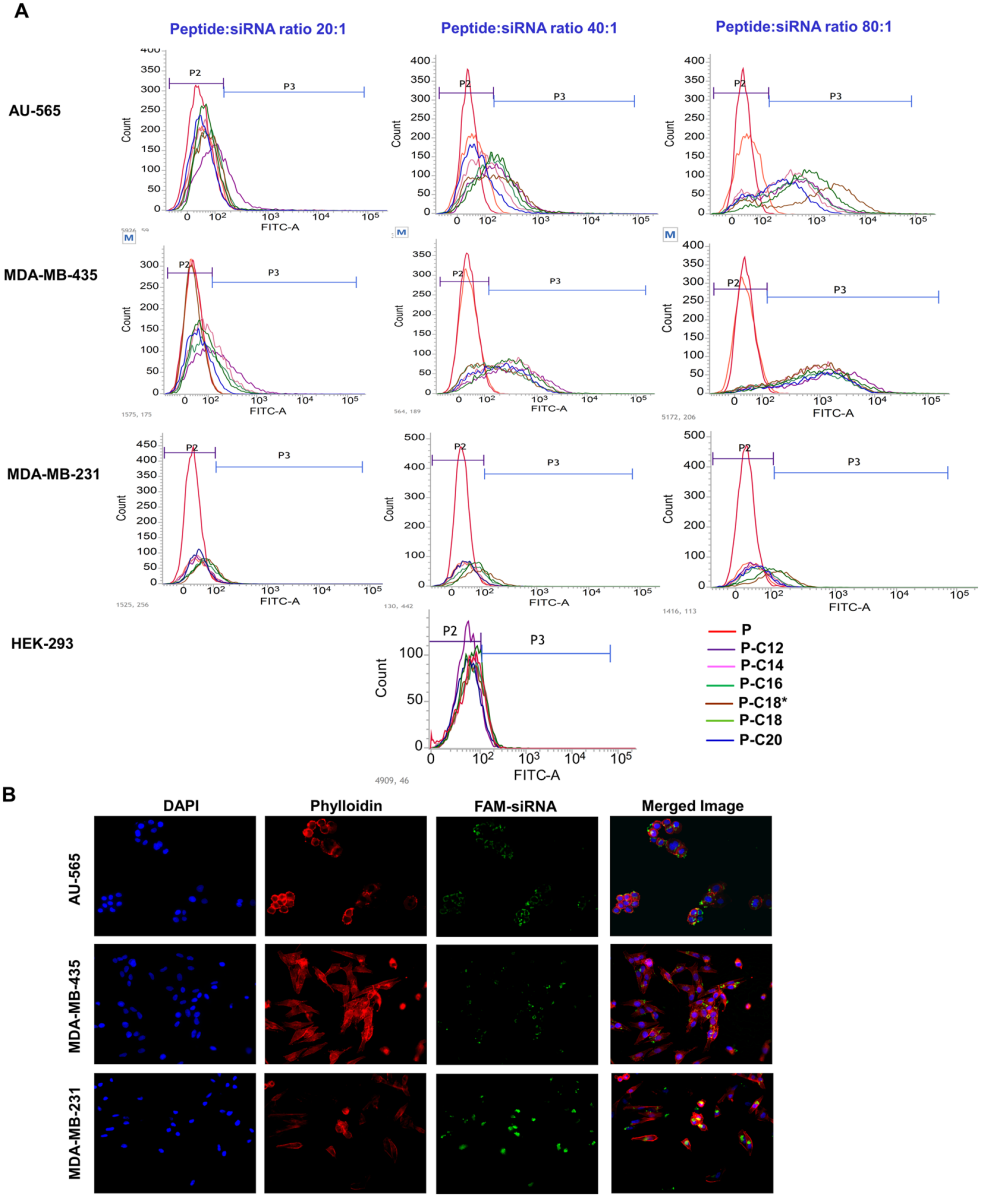
Visualization of uptake of FAM-siRNA. (**A**) Samples of the fluorescence signal for the cell populations for AU565, MDA-MB-435, and MDA-MB-231 cells after 24 h exposure to peptide:FAM-labelled siRNAs at w/w ratios of 20:1, 40:1, and 80:1 (N/P ≈ 27.3, 54.6, and 109, respectively, from left to right). Gates P2 and P3 represent fluorescence of un-treated and FAM-labelled siRNA treated cells, respectively. (**B)** Fluorescent microscope images (KEYENCE BZ-X700 all-in-one Fluorescence Microscope; Itasca, IL, USA) demonstrating (from left to right) DAPI-stained nuclei, Texas Red Phalloidin-stained cell membrane, FAM-labelled siRNA, and the merged images from AU565, MDA-MB-435, and MDA-MB-231 cells, after 24 h exposure to complexes formed with P-C18* peptide:FAM-labelled scrambled siRNA with w/w ratio of 80:1 (N/P ≈ 109).

Samples of the cell populations and the recorded fluorescence graphs are also presented in Fig. 7A. Fluorescence microscopy was also used to confirm the intracellular delivery of FAM-labeled siRNA in different cell lines. Our previous experiments with confocal microscope has confirmed the intracellular localization of the delivered siRNA in cytoplasm^11^. We selected the oleic acid-conjugated peptide (P-C18*) for these experiments, due to consistent efficiency for cellular internalization of siRNA in all the three cancer cell lines. Images are presented with blue (for DAPI used to stain the nucleus), red (for Texas Red Phalloidin used to stain cell membrane), and green (for FAM label conjugated to the scrambled siRNA) channels separately, and merged (Fig. 7B). The images confirmed significant fluorescent signal for labeled siRNA inside the cytoplasm of all three cancer cell lines.

### Silencing Efficiency

**Figure 8:**
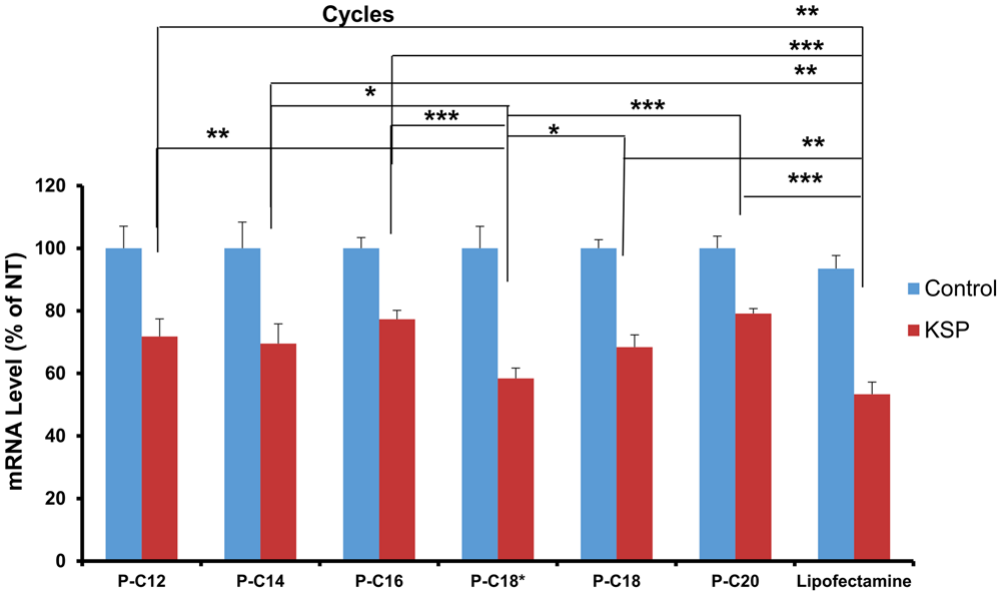
Quantification of down-regulation of KSP mRNA. Real-time PCR analysis of KSP expression (normalized based on beta actin) in AU-565 cells treated with scrambled (control) or KSP siRNA represented as mean + SD (n = 3), after 48 h of exposure to peptide/siRNA complexes (w/w ratio of 80:1; N/P ≈ 109) prepared with the modified peptides. The asterisks (*, **, ***) represent significant difference at levels of P < 0.05, 0.01, and 0.001, respectively.

The functional performance of the fatty acid-conjugated peptides was evaluated based on the silencing efficiency of a model protein (Kinesin spindle protein; KSP) as evaluated by real-time PCR. A sample of amplification graphs observed is shown in Fig. 8A. The silencing experiments were conducted in AU-565 cells, with all the modified peptides with peptide:siRNA ratio of 80:1 (N/P ≈ 109). The level of targeted mRNA (normalized based on the selected endogenous gene, β-actin) was significantly lowered via siRNA delivery by all the modified peptides, compared to cell exposed to complexes formed by the corresponding peptide and scrambled siRNA (Fig. 8B). The most significant effect was observed with PC-18* (RQ ≈ 0.58), where the mRNA level was significantly lower (with different P values) than other modified peptides.

## Discussion

This study was designed to analyze the effect of the hydrophobic modification on the characteristics of CGKRK peptide for siRNA delivery. This pentapeptide (Cys – Gly – Lys – Arg – Lys) has been shown to target tumor vasculature in glioblastoma^28^, breast cancer^29^, and an epidermal carcinogenesis animal model^25^. CGKRK has been used as the targeting moiety in polyethylene glycol – poly(ɛ-caprolactone) (PEG-co-PCL) nanoparticles to target tumor angiogenic blood vessels and human U87MG tumor cells^24^. Linked with PEG, it has also been studied as the targeting moiety for an adenoviral vector used for cancer gene therapy^30^. It has been reported that fatty acid conjugation to the therapeutic agents (e.g. protein, peptides, and siRNA) helps in improving the stability of the therapeutic agents in the blood^31–33^. We selected a range of different fatty acids for conjugation to CGKRK. Our previous experience indicates that smaller fatty acids might lack the significant impact on the carrier efficiency, and larger than 20 carbon chain length might limit the water solubility significantly, which in turn could negatively impact the efficacy of the delivery^12^. Therefore we selected fatty acid ranging from 12 to 20 carbon chain length. Fatty acylation was achieved on solid phase during peptide synthesis using mild condition in a quantitative yield. All the peptides were purified by using reverse phase HPLC and characterized by higher resolution MALDI mass spectrometer (see supplementary information). The peptides used in the study had > 95% purity.

Without the proposed hydrophobic modification, CGKRK did not readily interact with siRNA. A reduction in the size of complexes and a gradual shift in the surface charge from negative to positive values are among the signs of the ionic interaction between the carrier and siRNA, which were both observed for all of the modified peptides (Fig. 3A and B). The significant drop in particle size with changing the peptide:siRNA ratio from 1:1 (N/P ≈ 1.4) to 5:5 (N/P ≈ 6.8) indicates condensed nucleotides associated with the complex formation, while the positive ζ-potential is a sign of incorporation of more positively charged peptides into the complex, which shifts the overall surface charge of the particle. Although a size of ~100 nM seems to be an optimal size for delivery systems, effective *in vitro* uptake has been reported for particles as large as 200 nM or higher^7^. It has been reported that hydrophobic modification of siRNA carriers could increase the overall ζ-potential of the complexes. We speculate that a possible explanation for this observation is the hydrophobic barrier that forms with fatty-acid conjugated carrier. The ζ-potential is not quantified at the actual surface of the particle, but at the tightly bound layer, where the ions in the aqueous solution neutralize some of the electrical charge of the particle. Therefore, a hydrophobic component could form a barrier at the tightly bound layer, preventing this neutralization of charges, which would result in a more positive ζ-potential for the complexes formed with the hydrophobically modified peptide. Regardless of the mechanism, this positive ζ-potential would also further contribute to the higher cellular internalization efficacy with modified peptides.

This lack of proper interaction between unmodified peptide and siRNA was also confirmed by our binding affinity and serum stability experiments. While all conjugated peptides approached complete siRNA binding at peptide:siRNA ratios of 1:2.5 to 1:5, the siRNA binding affinity of the unmodified peptide plateaued well below values observed for modified peptides. We have previously reported a slight decrease in binding affinity of low molecular polyethyleneimine (PEI; with significant binding affinity to nucleotides) as a result of hydrophobic modification^12^, which seems to be expected due to increasing in hydrophobicity of the carrier. Despite initial affinity observed for the unmodified peptide in lower peptide:siRNA ratios, the binding was never completed. A slight increase was observed in the calculated BC50s (the peptide:siRNA ratio required for 50% binding), and P-C20 showed the highest BC50, which indicates the lowest affinity with the most hydrophobic modification. However, no significant correlation between BC50 and the size of the fatty acids was observed, mostly due to the unexpected high affinity of peptides modified with fatty acids with 18 carbon chain length (PC-18 and PC18*; Fig. 3D).

siRNA is instantly degraded *in vivo* (or even in cell culture) by RNase A-type nucleases^34^. In fact, this susceptibility to enzymatic degradation plays a major role in the extremely short serum half-life for siRNA, which has been reported as less than 30 min^35^. Therefore, one of the characteristics of an efficient siRNA carrier is its ability to protect siRNA from early degradation. Based on the ζ-potential quantifications, we expected significant protection against enzymatic degradation for all the modified peptides. An overall positive charge for the peptide/siRNA complexes indicates the complete binding, neutralization of negative charge, and condensation of siRNA, which indicates completion of the complex formation. The condensed complexes are expected to withstand exposure to degrading enzymes for extended periods of time. We validated the efficiency of our serum stability test by negative (exposing free siRNA to saline) and positive (exposing free siRNA to serum) controls, which confirmed full stability in the absence of serum and efficient enzymatic degradation in the presence of serum, respectively. As expected by negative ζ-potential and incomplete binding, unmodified peptide offered no detectable protection against enzymatic degradation, similar to free siRNA (Fig. 4). On the other hand, our all fatty acid-modified peptides protected siRNA from the serum degradation. This is similar to the results reported by Li Y *et al*.^36^, which indicates improvement in stability and binding affinity of glucagon like peptide-1(GLP-1) after conjugation with the fatty acid-like molecules. Although all the peptide conjugates offered protection against the serum degradation at highest peptide:siRNA ratio, P-C20 was the only modified peptide that offered any significant protection at the lowest peptide:siRNA ratio tested (5:1; N/P ≈ 6.8), which could be explained by the highest hydrophobicity (and the least interaction with the aqueous environment) of this carrier among the studied peptides.

The success of many promising siRNA delivery systems has been marred due to unacceptable toxicity. It has been speculated that potential toxicity of positively charged siRNA carriers could be due to transient nano-sized holes in the cytoplasmic membrane, which could also be partially responsible for enhanced cellular uptake^37^. Complex formation, on the other hand, will change the overall surface charge of the particles interacting with the cell membrane, due to partial neutralization of the charges. For that reason, we selected two approaches to evaluate the potential toxicity of the synthesized peptides: first, exposing the cells to peptides, and second exposure of cells to peptide/siRNA complexes. We also selected four different cell lines (three cancer cell lines, plus HEK293 cells) to evaluate the consistency of the results in a wider range of cell types. In this study, however, neither a significant toxicity nor a trend was observed for any of the peptides in the selected cell lines, via these two approaches. This was expected since no significant cytotoxicity has been reported for the unmodified peptide.

The site of action for siRNA is cytoplasm, and therefore, cellular internalization is a crucial step for reliable and reproducible silencing efficiency. However, free siRNA has little chance for significant internalization due to its chemical characteristics. Many different strategies have shown promising results in siRNA cellular uptake, including cell binding ligands and using hydrophobic components (which was the basis for our hydrophobic modification hypothesis). Although active targeting moieties, including CGKRK, have been mainly used to enhance the accumulation of their cargo in the target tissue or cells, they may also increase the cellular uptake of siRNA by enhancing interaction with the cell membrane, which seems to be required for effective internalization. For instance, a study in NOD/SCID mice showed comparable accumulation of siRNA in Neuro2 xenograft tumor with targeted and non-targeted nanoparticles; however, the silencing efficiency was almost doubled with the use of transferrin as the targeting ligand, which indicates an enhanced uptake^38^. The same beneficiary effect has been reported for K_16_GACYGLPHKFCG peptide in cationic liposomes^39, 40^, folate in polymeric delivery systems^41, 42^, and “mannosylated” polymer that interacts with mannose receptors^43^. While free siRNA did not show any detectable internalization in any of the selected cell lines as expected, it was interesting that complexes formed with unmodified peptide did not improve the uptake either (Figs 6 and 7). This is, however, explained by the incomplete and inefficient complex formation, negative ζ-potential, and the lack of serum stability for siRNA delivered with unmodified peptide. The modified fatty acid-conjugated peptides, on the other hand, showed significant improvement in siRNA uptake in all selected cancer cell lines, which continually increased with higher peptide:siRNA ratio. We have previously reported a similar pattern for the uptake of siRNA complexes with hydrophobically-modified PEIs with different polymer:siRNA ratios^12, 44, 45^. No specific trend was observed for the effect of fatty acid size on the siRNA uptake. It is noteworthy that none of the studied peptides induced significant internalization in the non-tumorigenic HEK293 cells. CGKRK peptide was discovered after multiple rounds of phage display^25^, which specifically binds to the neurovascular endothelial cells using heparan sulfate receptor. The expression of this receptor is little to none in the HEK293 cells^46^ due to non-malignant nature of these cells. Heparan sulfate receptors are sulfate polysaccharides, which present in neovascular endothelial cells and tumor cells specifically^26^.

Cellular internalization does not guaranty effective silencing. Depending on the route of internalization, particles could be trapped and degraded on their way to the cytoplasm. For example, in the case of endocytosis, siRNA/carrier needs to escape the endosome by non-contact mechanisms (that could involve endosomal eruption; e.g., “proton sponge effect”)^47^, or direct interaction of the carrier with the endosomal bilayer membrane. Also, after entering the cytoplasm, the siRNA would only be functional if it is released from the carrier in a timely manner. In fact, the disassembly of the carrier/siRNA package could become a major barrier for efficient silencing^39, 48^. Therefore, our final step in this project was to demonstrate effective interference with the selected mRNA in a cancer cell line. We selected kinesin spindle protein (KSP) for this purpose, due to its relevance to cancer therapy. This protein is involved in centrosome separation and bipolar spindle assembly, and silencing KSP has shown positive effect on suppression of subcutaneous melanomas and ovarian tumors^49^. An effective down-regulation of KSP mRNA levels was observed for all modified peptides in the real-time PCR analysis (Fig. 8). This effect was the most significant for P-C18*, which showed the most efficient cellular internalization in AU565 cells as well. Interfering with KSP mRNA with Lipofectamine® 2000, as a widely used commercial siRNA transfecting agent, resulted in a RQ of 53.3, which was not statistically different than the efficiency of P-C18*. This observation confirms our findings in cellular internalization analysis and the proper cytoplasmic delivery of siRNA by oleic acid-conjugated CGKRK peptide.

## Conclusions

In this study, we have demonstrated the development of a tumor-targeted delivery system using fatty acylation of CGKRK peptide. Fatty acylation was carried out using various fatty acids (C12 to C20 including unsaturated C18 oleic acid) during peptide synthesis with excellent yield. The characterization studies revealed complex formation with siRNA for all modified peptides, which showed significant binding affinity to siRNA and promising capability in protecting siRNA against early degradation. Safe and effective intracellular delivery of siRNA was also confirmed by cytotoxicity and cellular uptake experiments, respectively. However, no significant cellular uptake was detected in human non-tumorigenic cells for any of the modified peptides, which is important to confirm tumor specificity of the designed delivery systems. Efficient interference with the mRNA of an important protein involved in cancer cell proliferation indicates potential applications of the fatty acid-conjugated CGKRK as a tumor-specific siRNA carrier.

## Electronic supplementary material

Supplementary Information
