# Supplementary material for: Tumor-targeted delivery of siRNA using fatty acyl-CGKRK peptide conjugates
Source: Sci Rep. 2017 Jul 21;7:6093. doi: 10.1038/s41598-017-06381-y (PMC5522445; doi:10.1038/s41598-017-06381-y)
Supplement: Supplementary file 1 — Supplementary Information [file 41598_2017_6381_MOESM1_ESM.docx]

**Supporting information**

**Tumor-targeted delivery of siRNA using fatty acyl-CGKRK peptide conjugates**

Meenakshi Sharma^1^, Naglaa Salem El-Sayed^1,2^, Hung Do^1^, Keykavous Parang^1^, Rakesh Kumar Tiwari^1*^, Hamidreza Montazeri Aliabadi^1*^

^1^Center for Targeted Drug Delivery, Department of Biomedical and Pharmaceutical Sciences, Chapman University School of Pharmacy, Harry and Diane Rinker Health Science Campus, Irvine, California 92618, United States

^2^Cellulose and Paper Department, National Research Center, Dokki 12622, Cairo, Egypt

*** Corresponding Authors**

*Rakesh Kumar Tiwari, Ph. D*

Chapman University School of Pharmacy

Harry and Diane Rinker Health Science Campus

#263, 9401 Jeronimo Road

Irvine, CA 92618, USA

Tel: (714) 516-5483. Fax: (714) 516-5481. E-mail: [tiwari@chapman.edu](mailto:tiwari@chapman.edu)

*Hamidreza Montazeri Aliabadi, Ph. D*

Chapman University School of Pharmacy

Harry and Diane Rinker Health Science Campus

#211, 9401 Jeronimo Road

Irvine, CA 92618, USA

Tel: (714) 516-5492. Fax: (714) 516-5481. E-mail: [montazer@chapman.edu](mailto:montazer@chapman.edu)

**Table of Contents Page**

1. MALDI Spectra of peptides used in this studies. S3

2. Cytotoxicity of peptides in HEK cells S6

1. MALDI Spectra of peptides used in this studies.


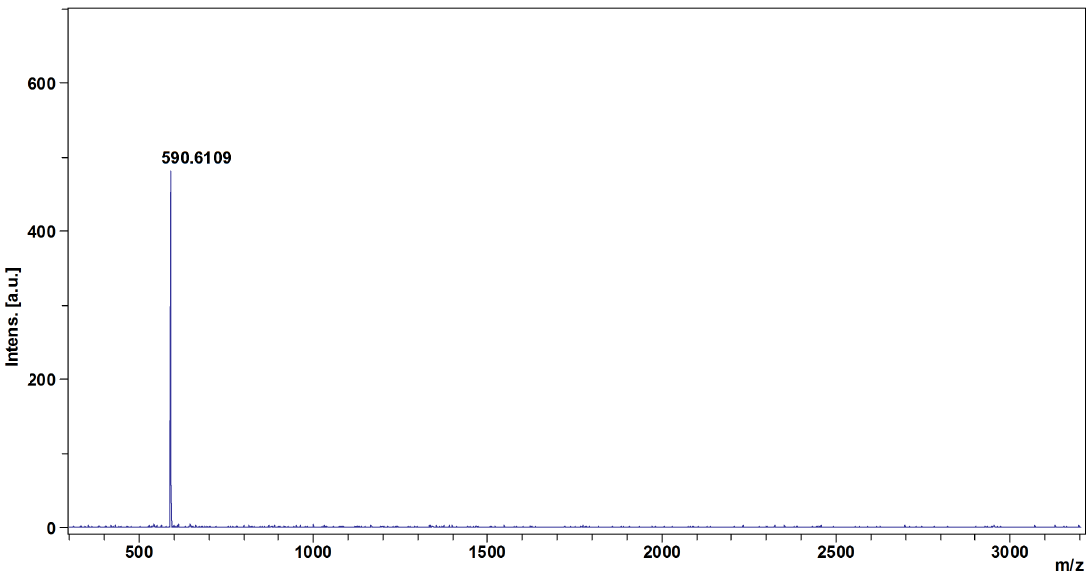


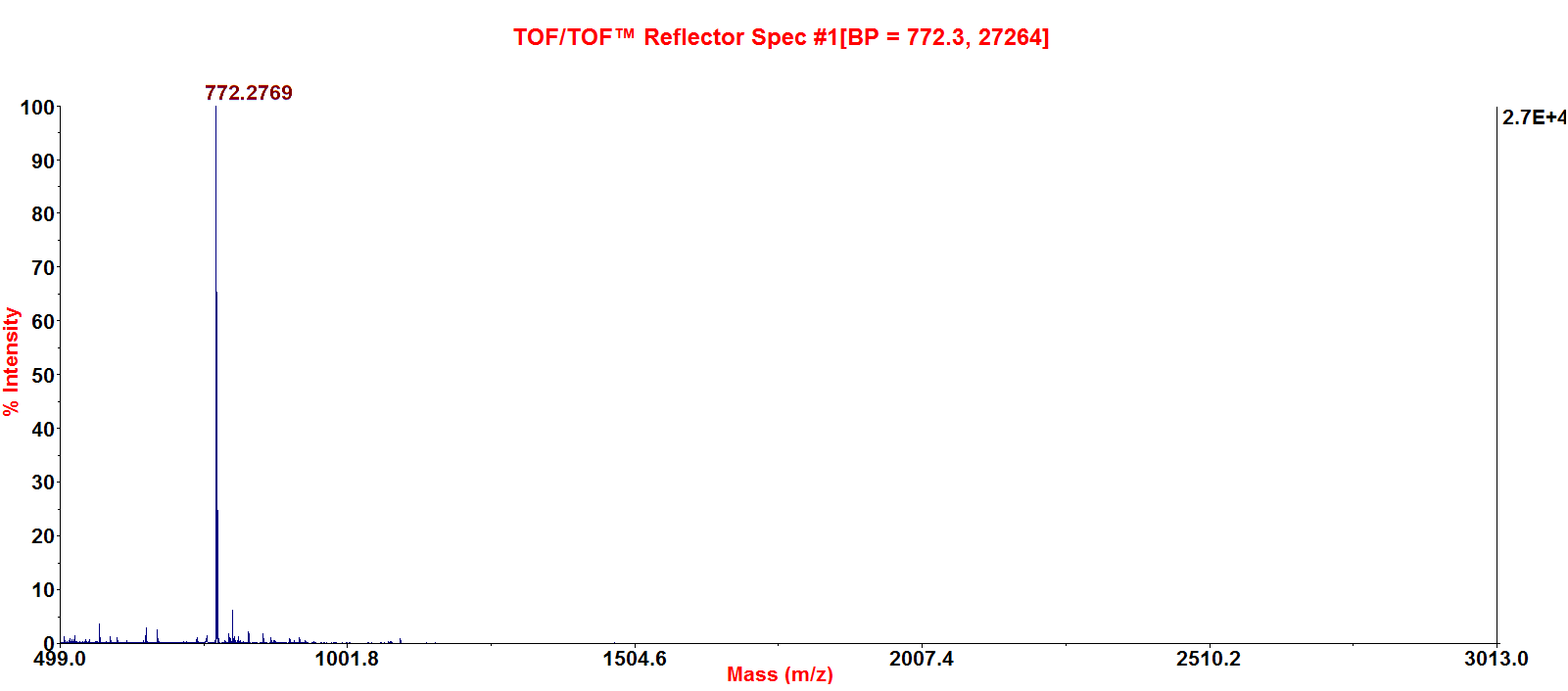


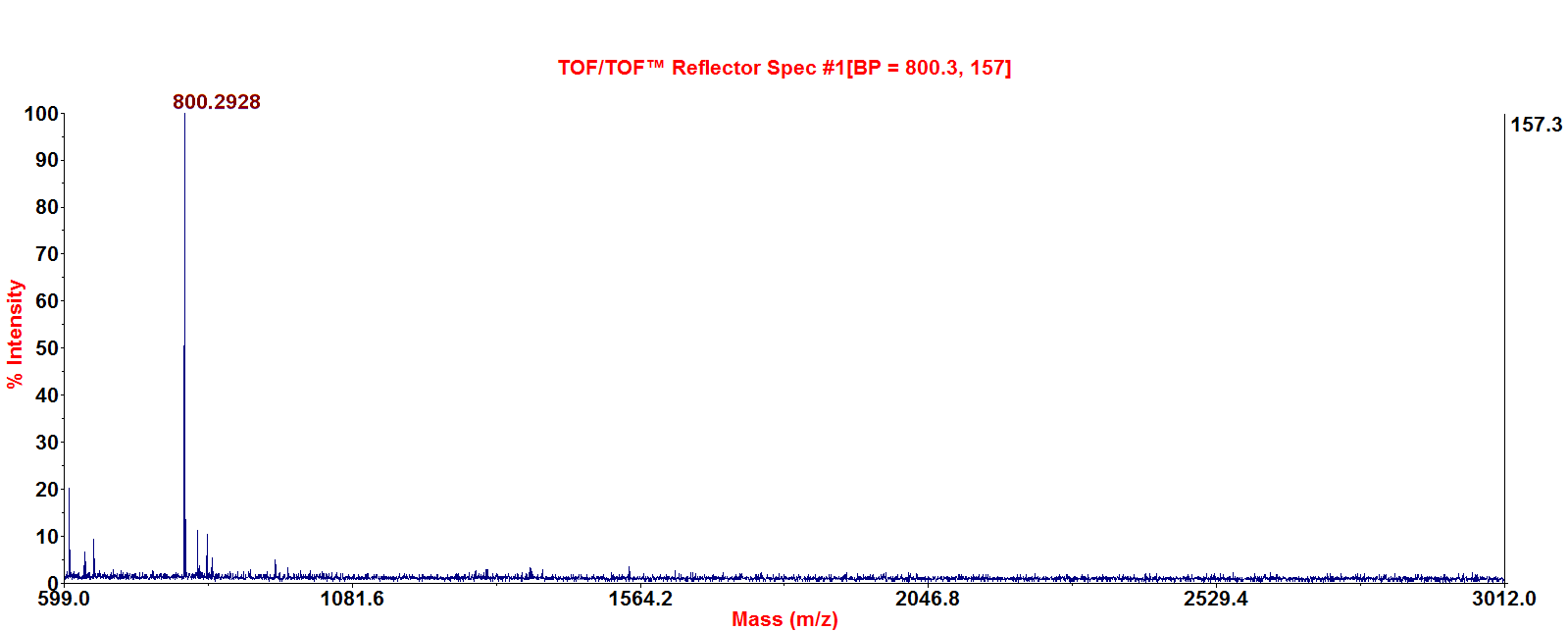


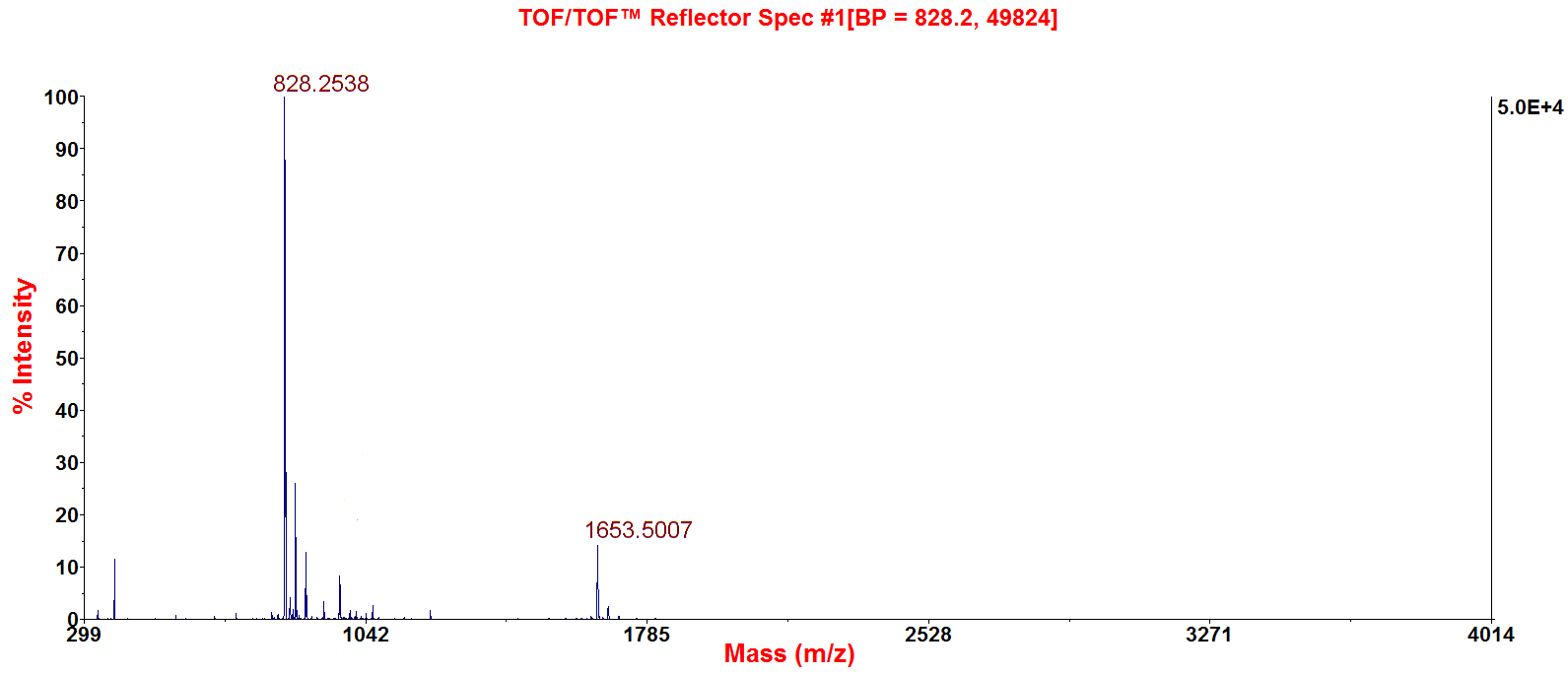


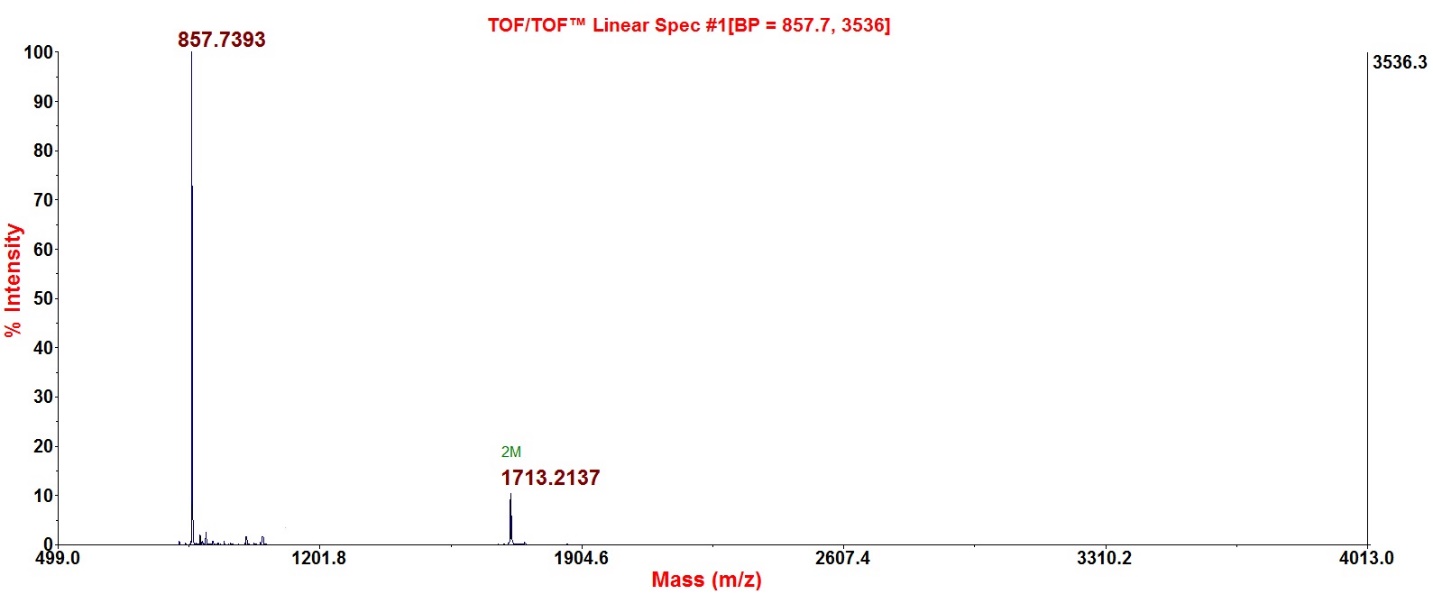


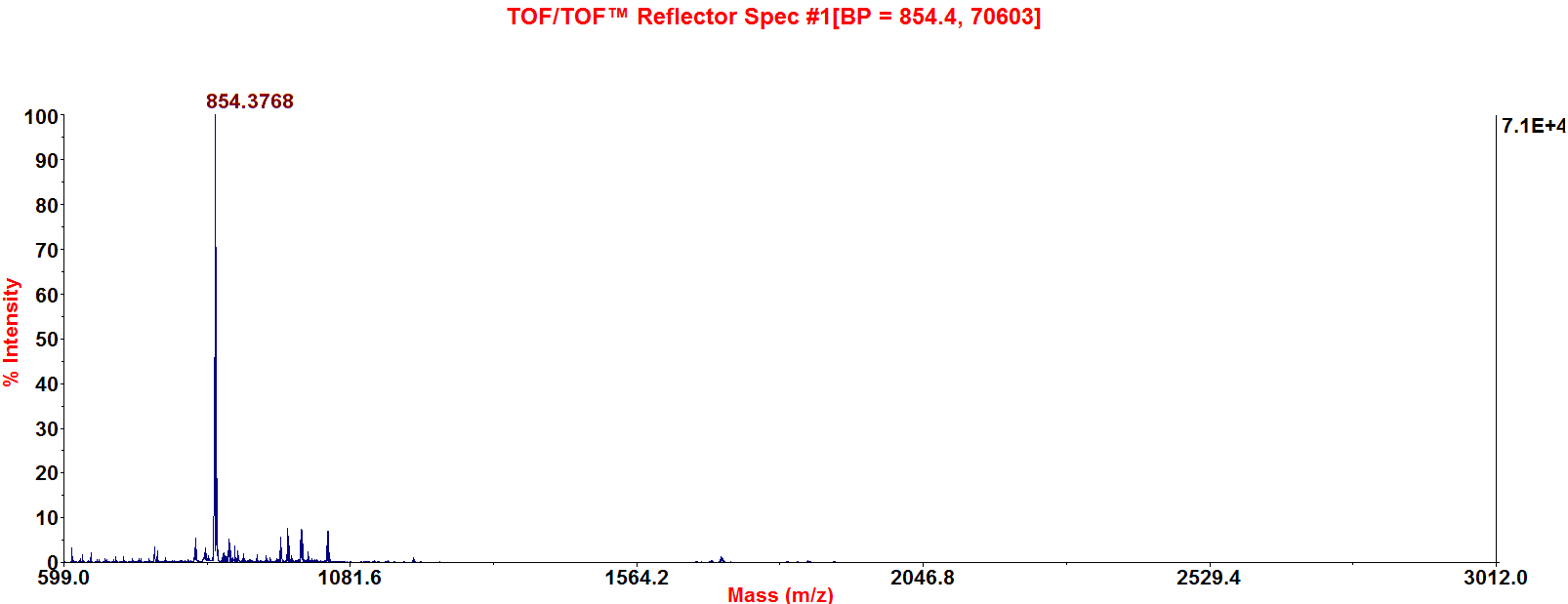


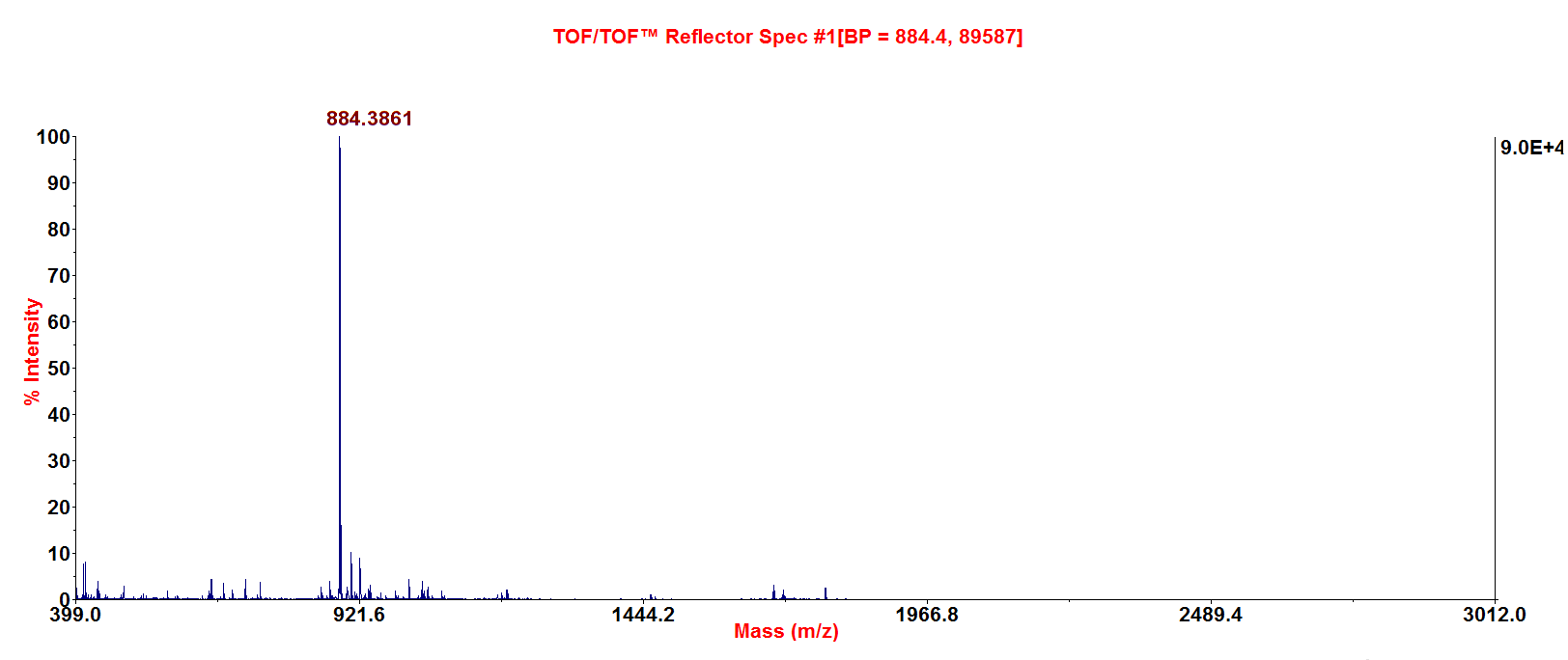


2. Cytotoxicity of peptides in HEK cells

**Figure S1**: Cytotoxicity of peptides in the HEK cells.
